# Combined Tumor Cell-Based Vaccination and Interleukin-12 Gene Therapy Polarizes the Tumor Microenvironment in Mice

**DOI:** 10.1007/s00005-015-0337-y

**Published:** 2015-03-24

**Authors:** Magdalena Jarosz-Biej, Ryszard Smolarczyk, Tomasz Cichoń, Natalia Kułach, Justyna Czapla, Sybilla Matuszczak, Stanisław Szala

**Affiliations:** 1 Center for Translational Research and Molecular Biology of Cancer, Maria Skłodowska-Curie Memorial Cancer Center and Institute of Oncology, Gliwice Branch, Wybrzeże Armii Krajowej 15, 44-101 Gliwice, Poland; 2 Department of Animal Physiology and Ecotoxycology, University of Silesia, Katowice, Poland

**Keywords:** Tumor cell-based vaccine, CAMEL, IL-12, Combined anti-tumor therapy, Polarization of tumor microenvironment

## Abstract

Tumor progression depends on tumor milieu, which influences neovasculature formation and immunosuppression. Combining immunotherapy with antiangiogenic/antivascular therapy might be an effective therapeutic approach. The aim of our study was to elaborate an anticancer therapeutic strategy based on the induction of immune response which leads to polarization of tumor milieu. To achieve this, we developed a tumor cell-based vaccine. CAMEL peptide was used as a B16-F10 cell death-inducing agent. The lysates were used as a vaccine to immunize mice bearing B16-F10 melanoma tumors. To further improve the therapeutic effect of the vaccine, we combined it with interleukin (IL)-12 gene therapy. IL-12, a cytokine with antiangiogenic properties, activates nonspecific and specific immune responses. We observed that combined therapy is significantly more effective (as compared with monotherapies) in inhibiting tumor growth. Furthermore, the tested combination polarizes the tumor microenvironment, which results in a switch from a proangiogenic/immunosuppressive to an antiangiogenic/immunostimulatory one. The switch manifests itself as a decreased number of tumor blood vessels, increased levels of tumor-infiltrating CD4^+^, CD8^+^ and NK cells, as well as lower level of suppressor lymphocytes (Treg). Our results suggest that polarizing tumor milieu by such combined therapy does inhibit tumor growth and seems to be a promising therapeutic strategy.

## Introduction

Tumor microenvironment participates in two strictly related processes crucial for tumor progression: formation of tumor blood vessels and presence of immunosuppression milieu, which enables cancer cells to escape from immune surveillance (Huang et al. 2013; Szala et al. 2010). This is because the cells that contribute to tumor microenvironment formation release proangiogenic agents which also act as immunosuppression stimulants (Facciabene et al. 2012; Szala et al. 2010; Tartour et al. 2011; Terme et al. 2012). Angiogenesis is critical for tumor development as tumors have to establish a blood supply to progress (Carmeliet and Jain 2011; Stockmann et al. 2014). Tumor microenvironment is regulated by numerous factors and processes (Hanahan and Coussens 2012; Swartz et al. 2012), as well as by immune system cells (e.g. T lymphocytes, dendritic cells (DCs), NK cells, or macrophages) (Ostrand-Rosenberg 2008; Shurin et al. 2012). These cells stimulate tumor growth by releasing proangiogenic and immunosuppressive factors (Tartour et al. 2011). Therefore, it appears reasonable to combine immunotherapy with therapies directed against tumor blood vessels (Huang et al. 2013; Szala et al. 2010). Certain drugs [e.g., anti-VEGFR2 monoclonal antibody (Li et al. 2006), sunitinib (Ozao-Choy et al. 2009)] destroy tumor blood vessels and trigger immune response by increasing the levels of CD4^+^ and CD8^+^ T lymphocytes, as well as by inhibiting the activity of immunosuppressive Treg or myeloid-derived suppressor cells (MDSC) (Szala et al. 2010). This causes polarization of the tumor microenvironment from a proangiogenic and immunosuppressive towards an antiangiogenic and immunostimulatory one (Ciomber et al. 2014; Huang et al. 2013; Jarosz et al. 2013; Ostrand-Rosenberg 2008).

The aim of our study was the elaboration of an anticancer therapeutic strategy based on induction of immune response which leads to polarization of tumor milieu. In our study, we investigated the effect of a tumor cell-based vaccine combined with murine interleukin (IL)-12 on immune response and polarization of the tumor microenvironment. The vaccine was constructed in our laboratory from B16-F10 melanoma cell cultures treated with CAMEL peptide. CAMEL, abbreviated as CA(1–7)M(2–9), consists of two fragments. One is derived from cecropin A [amino acids CA(1–7)], a peptide occurring in *Hyalophora cecropia* hemolymph, and the other from melittin [amino acids M(2-9)], a peptide from *Apis mellifera* (honeybee) (Smolarczyk et al. 2010). CAMEL peptide is capable of penetrating the cell membrane without damaging it. Following cell penetration, CAMEL localizes in mitochondria, inducing their swelling and consecutive disruption. The disruption of the mitochondrial membrane leads to a decrease in intracellular ATP level, as well as the release of HMGB1 (high-mobility group box 1 protein), triggering necrotic cell death (Smolarczyk et al. 2010). This peptide has not been used before as a tool to construct vaccines; however, in our previous studies, we showed that after intratumoral administration, CAMEL inhibited the growth of B16-F10 tumors (Smolarczyk et al. 2010, 2012). In this study, we used CAMEL as a cell necrosis-inducing agent. The lysates next served as a vaccine to induce an anticancer immune response. IL-12, as used in our study, was meant to further enhance the immune response. IL-12 was administered to animals in the form of gene therapy, and was mediated by plasmid DNA (Budryk et al. 2000; Ciomber et al. 2014; Jarosz et al. 2013). IL-12 is a pleiotropic immunomodulatory cytokine with antiangiogenic activity (Del Vecchio et al. 2007; Kilinc et al. 2006; Uemura et al. 2010). IL-12 increases the synthesis of interferon (IFN)-γ by NK and T cells, stimulates the growth and cytotoxicity of activated NK, CD8^+^ and CD4^+^ T cells, induces differentiation of CD4^+^ Th0 cells into Th1 phenotype, enhances antibody-dependent cell cytotoxicity against cancer cells, and induces IgG antibodies and inhibits the synthesis of IgE antibodies by B lymphocytes (Lasek et al. 2014). Additionally, IL-12 eliminates Treg lymphocytes from the tumor microenvironment, effectively abrogating tumor immunosuppression (Kilinc et al. 2006). IL-12 inhibits the formation of new blood vessels by stimulating antiangiogenic cytokines and chemokines. IL-12 also causes remodeling of the peritumoral extracellular matrix and tumor stroma, reprogramming of suppressor myeloid cells, and stimulates the overexpression of MHC class I molecules. All the above mechanisms are postulated to be responsible for the high potency of anti-tumor effects of IL-12 (Lasek et al. 2014).

In this work, we intended to investigate the effect of combination therapy on the tumor microenvironment. Our results suggest that this tumor cell-based vaccine, together with IL-12, induces immune response and polarizes the tumor microenvironment towards an antiangiogenic/antivascular and immunostimulatory one. Tumor milieu polarized in such a manner inhibits the growth of B16-F10 murine melanoma tumors in treated animals. It seems that the combination of tumor cell-based vaccine with IL-12 is a promising therapeutic approach that can be employed as one of the arms of multimodal anticancer strategies.

## Materials and Methods

### Mice, Plasmid, Drug and Cell Line

Mice (6- to 8-week-old, C57Bl/6NCrl females) were bred in our animal facility house. The experimental protocol was approved by the Local Ethics Commission (Medical University of Silesia, Katowice, Poland). Tumor growth inhibition was monitored using a murine B16-F10 melanoma model. Growing tumors were measured with calipers, and tumor volumes were determined using the formula: volume = width^2^ × length × 0.52. Plasmid pBCMGSNeo carrying a gene encoding murine IL-12 was obtained from Prof. H. Yamamoto (Osaka University, Japan). Plasmid preparations were isolated using a QIAGEN-Endo Free Giga Kit (QIAGEN GmbH, Hilden, Germany). CAMEL (KWKLFKKIGAVLKVL-NH_2_) and fluorescein isothiocyanate (FITC)-conjugated CAMEL were synthesized by Prof. W. Kamysz (Gdansk Medical University, Poland) using 9-fluorenylmethoxycarbonyl solid-phase chemistry. The purity of the synthesized peptide (95–97 %) was verified by reversed-phase HPLC. The physicochemical properties of CAMEL were further analyzed using matrix-assisted laser desorption ionization time-of-flight (MALDI-TOF) mass spectrometry (Smolarczyk et al. 2010). B16-F10 (murine melanoma) cell line (ATCC, Manassas, VA, USA) was maintained using RPMI 1640 medium (Gibco BRL, Paisley, UK) supplemented with 10 % fetal bovine serum (ICN Biomedicals, Costa Mesa, CA, USA). Cell cultures were kept under standard conditions (37 °C, 5 % CO_2_, 95 % humidity). B16-F10 (1.8 × 10^5^ CAMEL-treated cells and 3 × 10^4^ control cells) was used as in Casares et al. (2005).

### Tumor Cell Lysate Preparation

To generate a tumor cell-based vaccine, B16-F10 cells were treated with CAMEL peptide. Four doses (5, 10, 20 and 40 µM) of the peptide were checked. After 24 h the cells were stained with annexin V and 7-AAD and analyzed by flow cytometry (BD FACSAria™ III; BD, Franklin Lakes, NJ, USA). A 40 µM concentration of CAMEL was proven effective in killing over 97 % of cells. After washing twice with phosphate-buffered saline (PBS)^−^, the aliquots of 100 μL PBS^−^ containing lysate from 1.8 × 10^5^ CAMEL-treated tumor cells were used per animal. Vaccine samples were stored at −80 °C until use.

### Detection of Necrosis In Vitro

Necrosis of B16-F10 cells was determined using an Annexin V-PE Apoptosis Detection Kit (BD Pharmingen, San Diego, California, USA). Twenty-four hours after administering CAMEL detached B16-F10 murine melanoma cells (4 × 10^5^) were twice rinsed with PBS^−^ and resuspended in 1 mL of binding buffer. The staining procedure followed kit instructions. Analysis of labeled cells’ fluorescence was performed using an FACSAria™ III flow cytometer (BD). Additionally, B16-F10 cells (2 × 10^5^) were treated with FITC-conjugated CAMEL (40 µM), following which propidium iodide (PI; 0.5 mg/mL) was added. Images were taken using a Zeiss Cell Observer SD Semiconfocal Microscope (Carl Zeiss, Jena, Germany). Lens magnifications were 20× and 63×.

### Tumor Cell Challenge, Treatment with Immunomodulatory Factors

Seven days after inoculating the mice (lower flank) with B16-F10 melanoma cells (3 × 10^4^/100 μL PBS^−^), subcutaneous injections (contralateral flank) of CAMEL-treated tumor cell vaccine (1.8 × 10^5^/100 μL PBS^−^) were initiated. Tumor cell-based vaccine was administered three times, 1 week apart. Additionally, in the combined therapy regimen, 24 h following the administration of each tumor cell-based vaccine, plasmid DNA encoding IL-12 gene was injected at the same spot [50 µg/100 µL PBS^−^ pH 7.4 (Budryk et al. 2000; Mitrus et al. 2006)].

### Flow Cytometric Analysis

Mice were killed on the 28th day of the experiment. Left and right cervical lymph nodes (LNs) were isolated. LN cells were counted and single-cell suspension was used for flow cytometric analysis. Following live cell gating, the percentage of CD4^+^ and CD8^+^ T lymphocytes was determined. Also, tumor material was collected for flow cytometric analysis; single-cell suspension was obtained using a digestion mix (0.5 mg/mL collagenase A, Sigma Aldrich; 0.2 mg/mL hyaluronidase type V, Sigma Aldrich; 0.02 mg/mL DNase I, Roche; per 0.25 g of tumor tissue). Red blood cells were lysed using 0.15 M ammonium chloride (Sigma Aldrich, St Louis, MO, USA). Dead cells were removed by centrifugation using Lympholyte-M gradients (Cedarlane, Ontario, Canada). To identify the subpopulations of T lymphocytes, the following antibodies were used: PE-Cy7-CD3e, PE-CD4 and FITC-CD8a (BD Pharmingen). Treg lymphocytes were identified with FITC-CD4, APC-CD25 and PE-Foxp3 antibodies (eBiosciences; San Diego, CA, USA). Finally, to identify the level of NK cells, an anti-mouse CD49b (pan-NK cells) antibody was used (eBioscences). In flow cytometric analyses (BD FACSCanto, BD), gate dividing negative from positive cells was based on isotype antibody control probes (Jarosz et al. 2013).

### Immunohistochemistry

Mice were killed on the 28th day of the experiment. Tumors were excised to identify tumor vessels; paraffin sections (5 μm) were stained immunohistochemically: following overnight incubation (4 °C) with rabbit anti-CD31 polyclonal primary antibody (Abcam, Cambridge, UK), the sections were incubated (45 min/room temperature) with FITC-conjugated secondary antibody (Vector Laboratories, Burlingame, CA, USA) and cover slipped with Vectashield mounting medium containing DAPI (Vector Laboratories). Images were taken using a Zeiss LSM 710 confocal microscope (Carl Zeiss). The numbers of blood vessels in each group were determined based on 10 visual field counts from four tumor sections (lens magnification: 20×).

### Statistical Analysis

The statistical significance of differences between the experimental and control groups were evaluated by the analysis of variance test (ANOVA). *P* values <0.05 were considered statistically significant.

## Results

### CAMEL Peptide Induces Necrosis

Tumor cell-based vaccine was prepared following melanoma cell cultures’ treatment with CAMEL peptide. To achieve this, CAMEL concentration resulting more than 95 % of B16-F10 cells’ death was determined. Four different peptide doses (5, 10, 20 and 40 µM) were examined. Quantification of apoptotic/necrotic cells was performed by flow cytometry and PE-annexin V, as well as 7-AAD staining.

**Fig. 1:**
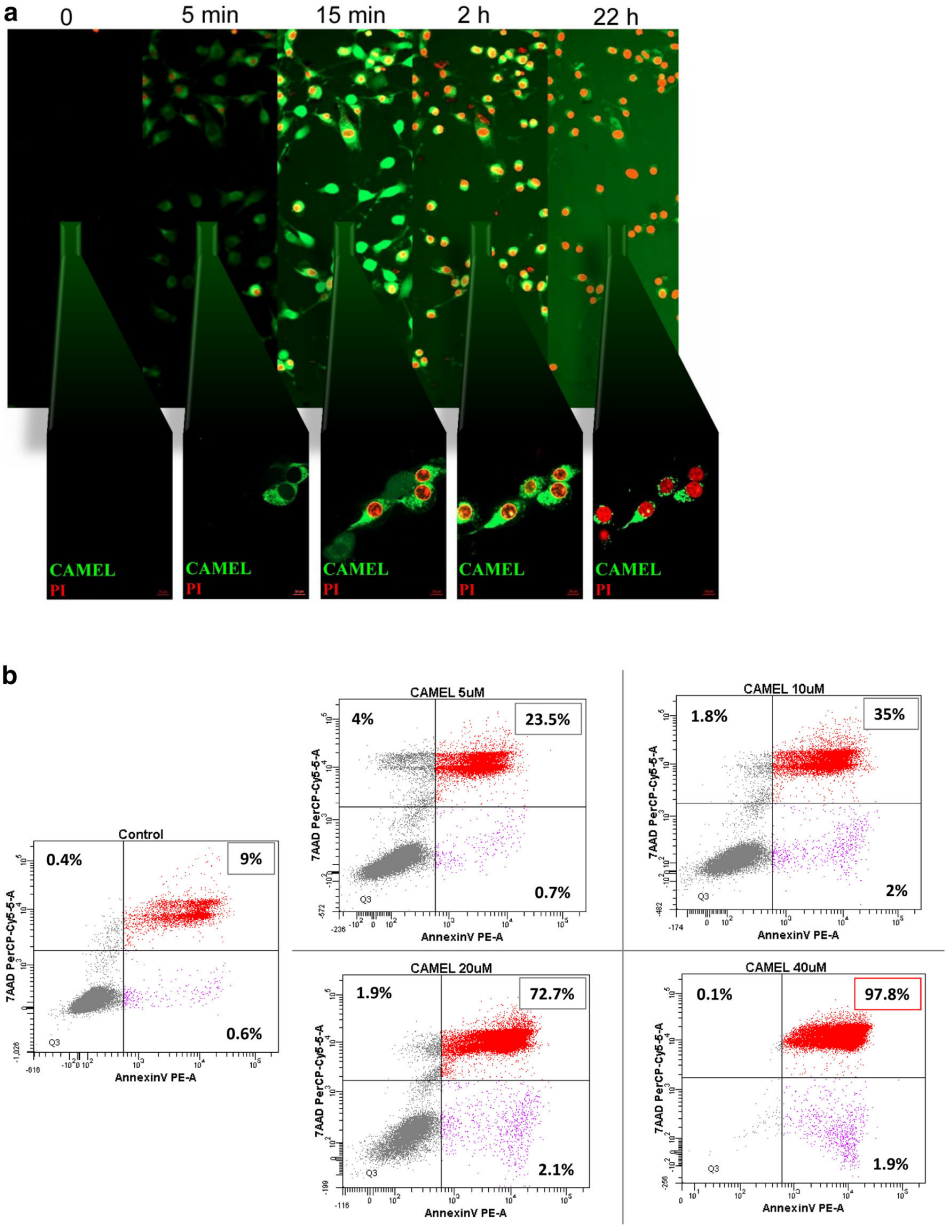
Tumor cell-based vaccine construction. Induction of necrosis by CAMEL. **a** B16-F10 cells were treated with FITC-CAMEL, and then propidium iodide (PI) was added. Lens magnification: ×20 and ×63. The peptide (*green fluorescence*) localizes in the cytoplasm. With time, the accumulation of CAMEL in cells causes cell swelling and rupture of the plasma membrane, triggering cell death (*red fluorescence*). **b** B16-F10 cells treated with CAMEL (5–40 µM). Twenty-four hours later cells were stained with annexin V and 7-AAD and analyzed by flow cytometry. After treatment with 40 µM CAMEL >97 % cells were necrotic (annexin V^+^7-AAD^+^)

We confirmed that CAMEL localizes inside B16-F10 murine melanoma cells and causes necrotic cell death. The peptide (FITC-CAMEL staining) does not cause the destruction of cell membranes (lack of red PI staining of cell nucleus) and localizes in the cytoplasm. With time, the accumulation of CAMEL in cells causes cell swelling and rupture of the plasma membrane triggering cell death (red PI staining of cell nucleus) [Fig. 1a; (Smolarczyk et al. 2010)]. Above 97 % of the cells underwent necrosis (annexin V^+^7-AAD^+^) as a result of the treatment with 40 µM CAMEL concentration (Fig. 1b). Accordingly, tumor cell-based vaccine was prepared from B16-F10 cell lysates following cells’ treatment with 40 µM CAMEL.

### Combination of Tumor Cell-Based Vaccine and IL-12 Effectively Inhibits Tumor Growth

**Fig. 2:**
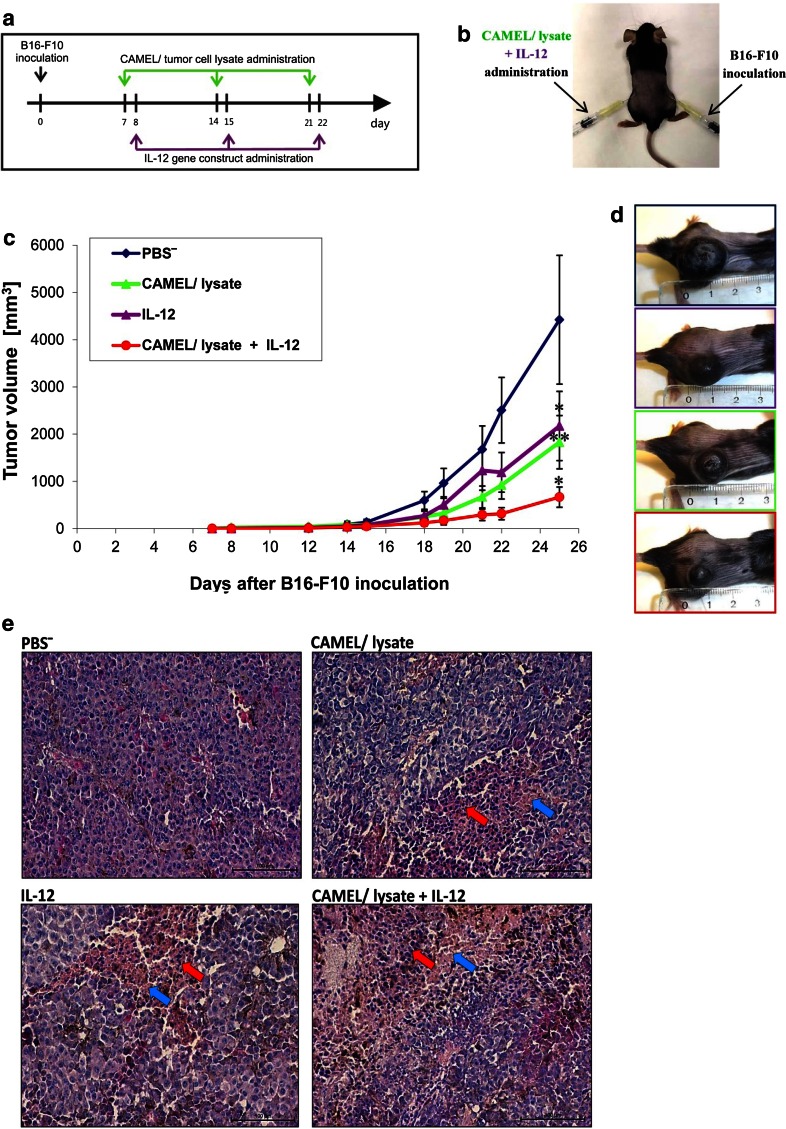
Inhibition of B16-F10 tumor growth in response to combination therapy involving tumor cell-based vaccine and IL-12. **a**, **b** Seven days after inoculation (lower flank) with B16-F10 melanoma cells (3 × 10^4^/100 μL PBS^−^; *n* = 9) subcutaneous injections of lysate from CAMEL-treated tumor cells (1.8 × 10^5^/100 μL PBS^−^) were started (contralateral flank). The vaccine was administered three times, 1 week apart. In combined therapy regimen, 24 h after each vaccine administration plasmid DNA encoding IL-12 gene was additionally injected at the same site (50 μg/100 μL PBS^−^). **c** Combined therapy was highly effective in inhibiting tumor growth compared to controls receiving single-agent therapy. Compared to control, statistical differences on the 25th day of therapy were *P  < 0.01, **P < 0.05. **d** Photographs taken on the 28th day of the experiment. **e** Tumors (*n* = 3) were collected 4 weeks after challenge and counterstained with hematoxylin/eosin. Considerable necrotic areas (*red arrows*) and immune cell infiltration (*blue*) seen in tumor sections from mice treated with tumor cell-based vaccine, IL-12, or their combination. Magnification: ×20

Next, we examined the therapeutic effect of our tumor cell-based vaccine and its combination with gene therapy mediated by plasmid DNA construct encoding murine IL-12. For this purpose, mice were first inoculated with B16-F10 cells (right flank) and, after 7 days, the tumor cell-based vaccines were administered subcutaneously (left flank) three times, 1 week apart (Fig. 2a, b). Each vaccine administration was followed 24 h later by gene therapy (injection in the same spot).

We noted that our tumor cell-based vaccine inhibits the growth of B16-F10 murine melanoma, compared to control (PBS^−^). However, significant inhibitory results were obtained using a combination of the vaccine with IL-12 (85 % inhibition of tumor growth) rather than using either of the agents alone (59 % inhibition following vaccine administration and 51 % inhibition in the case of gene therapy, see Fig. 2c, d). Comparing tumor-derived histochemical specimens from mice treated with the combination regimen and those from the control, we noted decreased number of tumor blood vessels, more extensive necrotic areas and enhanced infiltration of immune cells in tumor sections in the case of combined therapy (Fig. 2e).

### Tumor Cell-Based Vaccine in Combination with IL-12 Induces Immune Response

We examined the effect of tumor cell-based vaccine as well as IL-12 on immune response. One week after inoculating mice with cancer cells, the therapy was initiated with vaccine administration (on days 7, 14 and 21), followed by IL-12-mediated gene therapy (days 8, 15 and 22). One week after the last drug injection, mice were killed and cervical lymph nodes as well as tumors were collected. The levels of T lymphocytes and NK cells were determined by flow cytometry.

**Fig. 3:**
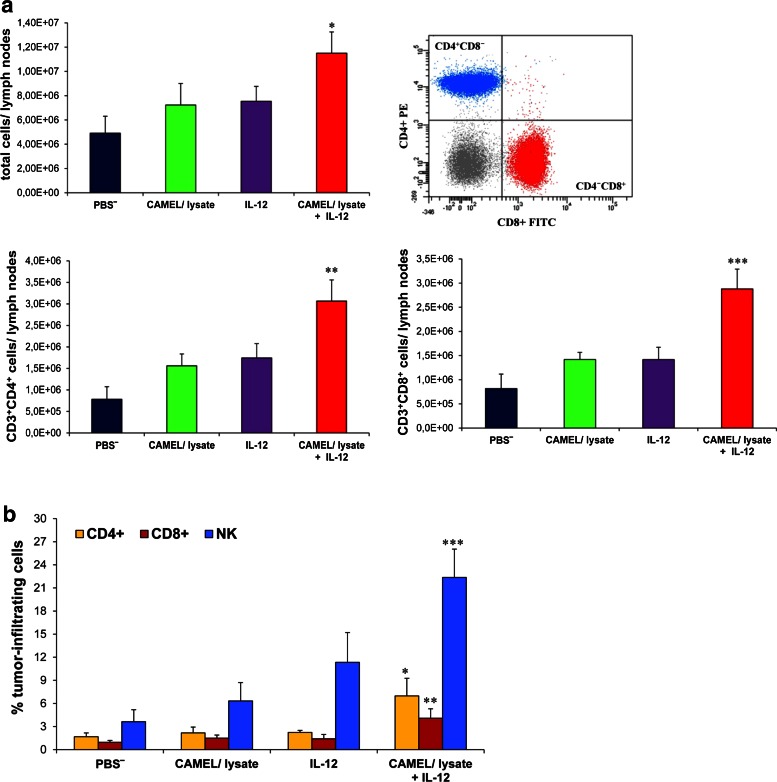
Induction of immune response by treatment with tumor cell-based vaccine and IL-12. One week after final drug injection, mice (*n* = 8) were killed. Cervical lymph nodes and tumor material were collected for flow cytometric analysis to determine the levels of T lymphocytes and NK cells. **a** Higher level of T cells in draining lymph nodes were noted in the case of combined therapy. Compared to controls, statistical differences were *P < 0.05, **P < 0.0025, ***P < 0.0005. **b** Significantly higher levels of tumor-infiltrating CD4^+^, CD8^+^ T cells and NK cells were found after combined therapy. Compared to controls, statistical differences were *P < 0.025, **P < 0.035, ***P < 0.02

The combinatory therapeutic regimen used resulted in the induction of both nonspecific and specific immune responses. This consisted of increased infiltration of CD4^+^ and CD8^+^ T lymphocytes in cervical lymph nodes of the treated mice (Fig. 3a). Also, a threefold increase of tumor-infiltrating CD4^+^ T cells and a twofold increase of CD8^+^ T cells, respectively, were observed as compared to monotherapy. CD4^+^ T cells play a central role in regulating all antigen-specific immune responses, and a role in both the induction and the effector phases of the anti-tumor response. CD8^+^ T cells can induce the cytolytic death of target tumor cells or promote tumor destruction via the secretion of effector cytokines such as IFN-γ or tumor necrosis factor (TNF)-α (Savage et al. 2014). In addition, we noted an increased expression level of CD49b, an NK cell marker, in tumors of the treated mice as compared to controls (twofold and threefold increase with respect to monotherapies and sixfold increase compared to control (PBS^−^), Fig. 3b). Natural killer (NK) cells are effector lymphocytes of innate immunity and provide a crucial contribution in tumor immunosurveillance (Waldhauer and Steinle 2008).

### Combination Therapy (Tumor Cell-Based Vaccine + IL-12) Decreases the Number of Tumor Blood Vessels and the Level of Tumor Treg Lymphocytes

After revealing an induced immune response following combination therapy, we examined its effect on the number of tumor blood vessels, as well as the level of tumor regulatory T lymphocytes. One week after the cessation of therapy tumor material was excised for immunohistochemical analysis. The staining was performed using an antibody directed against a marker of endothelial cells, CD31, as well as for cytofluorimetric assessment of the level of tumor-infiltrating Treg lymphocytes (CD4^+^CD25^high+^Foxp3^+^).

**Fig. 4:**
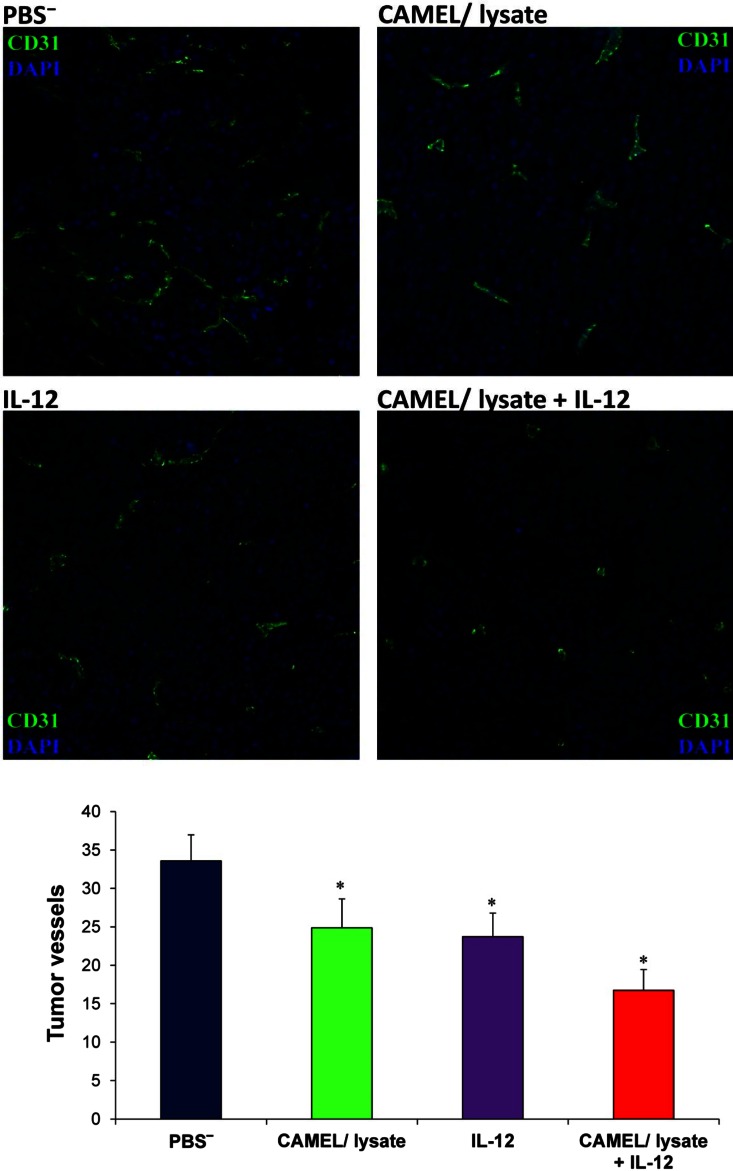
Reduced number of tumor blood vessels following combined therapy. One week after the last drug injection mice were killed, and tumors excised, fixed and stained with antibody against CD31 (marker of endothelial cells, *green fluorescence*). The number of vessels was counted for each experimental group in ten visual fields from four tumor sections (magnification: ×20). Significantly decreased numbers of vessels were found in tumor sections from mice treated with combined therapy as compared to controls. Compared to control, the statistical difference was *P < 0.0001

Immunohistochemical analyses demonstrated a significant reduction in the number of blood vessels in tumor specimens from mice treated with tumor cell-based vaccine combined with IL-12 (Fig. 4), when compared to controls (1.5-fold decrease with respect to monotherapies and twofold decrease compared to PBS^−^). Blood vessels play an important role in tumor progression. Solid tumors larger than 1–2 mm^3^ require their own vascular system for further progression (Folkman 1971; Tabi and Man 2006). Angiogenesis enables the supply of oxygen and growth factors to tumor cells and their microenvironment, and the removal of metabolites (Baeriswyl and Christofori 2009).

**Fig. 5:**
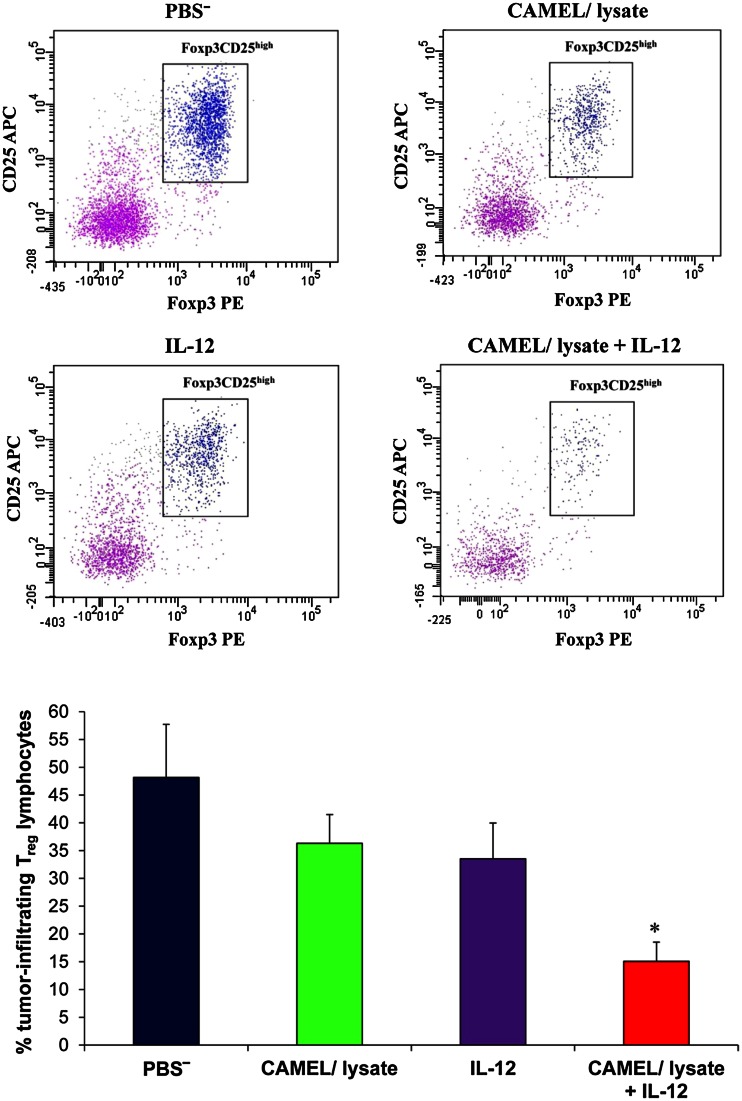
Reduced level of Treg lymphocytes after combined therapy. On the 28th day of the experiment, tumors (*n* = 8) were excised. Single-cell suspensions obtained were then used to quantitate Treg lymphocyte levels. The percentage of Foxp3^+^CD25^high+^ regulatory lymphocytes (subpopulation of CD4^+^ T lymphocytes) was determined from the lymphocyte population gate. The largest decrease in the level of tumor Treg lymphocytes was found for the group of mice treated with combined therapy. Compared to controls, the statistical difference was *P < 0.03

Cytofluorimetric analyses showed that monotherapies markedly decreased the level of tumor Treg lymphocytes. However, compared to the control (mice receiving PBS^−^) and monotherapies, more than threefold and twofold decrease was observed for Tregs level in tumors from mice treated with combination therapy. Regulatory T cells (Tregs) inhibit a cytotoxic immune reaction and promote tumor cell growth, angiogenesis and metastasis (Gutkin and Shurin 2014). Accumulation of Tregs in the tumor microenvironment shifts the balance between effector and suppressor lymphocytes, and induces an immunosuppressive state (Rabinovich et al. 2007; Zou 2005). Abrogation of the immunosuppressive state may be associated with the decreased number of Treg cells. This clearly underscores the benefit of a combined therapeutic approach in diminishing immunosuppression in the tumor microenvironment (Fig. 5).

**Fig. 6:**
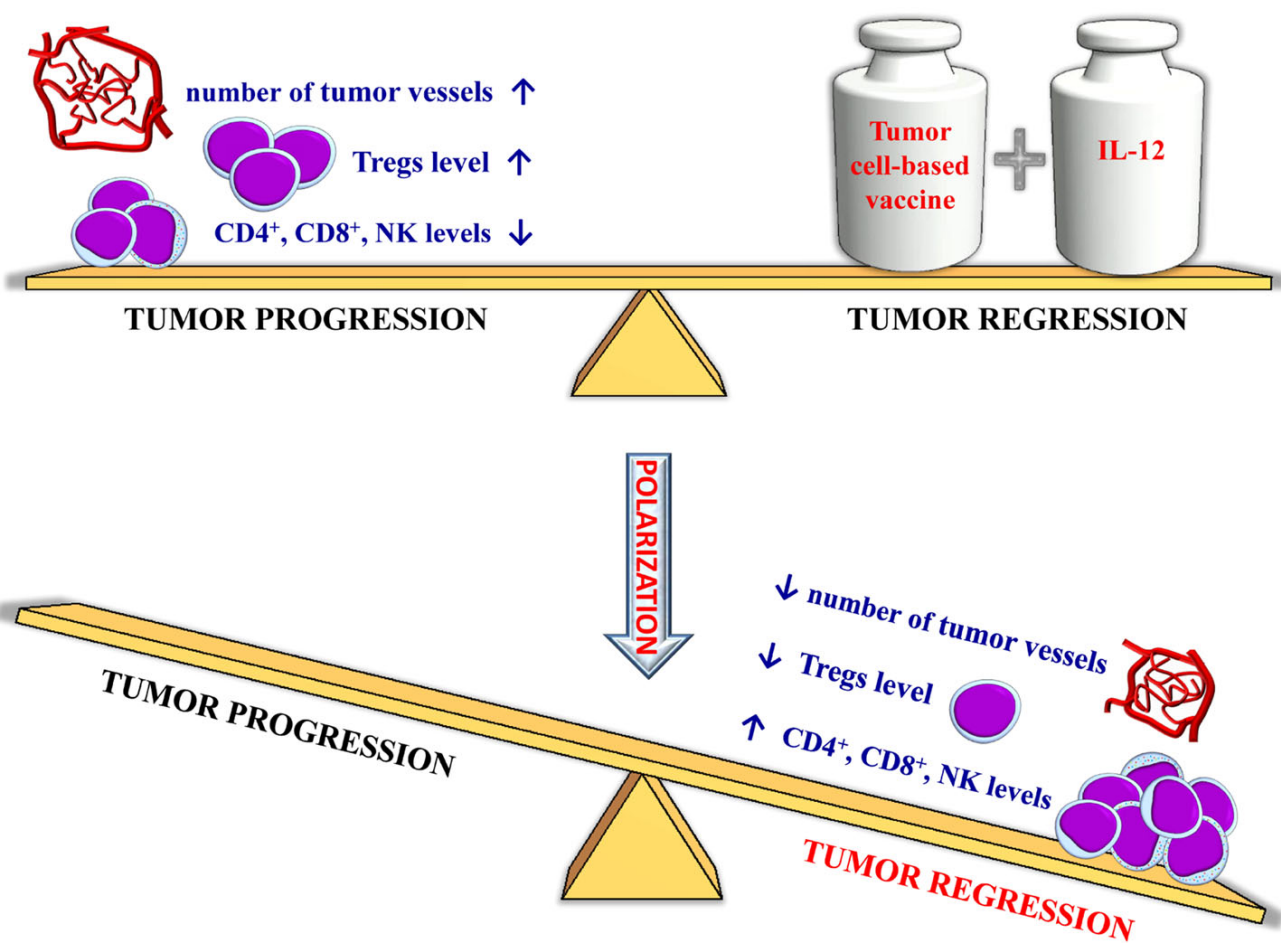
Polarization of tumor microenvironment by tumor cell-based vaccine and IL-12. Tumor progression depends on tumor milieu, which influences neovasculature formation and immunosuppression (allowing cancer cells’ escape from immune surveillance) (Hanahan and Coussens 2012; Szala et al. 2010). Combining immunotherapy with antiangiogenic therapy might be an effective therapeutic approach (Huang et al. 2013; Tartour et al. 2011). The combination tested seems to polarize the tumor microenvironment, resulting in a switch from a proangiogenic/immunosuppressive to an antiangiogenic/immunostimulatory one. The switch appears as a decreased number of tumor blood vessels, increased levels of CD4^+^, CD8^+^ T cells and NK cells, as well as lower levels of suppressor lymphocytes (Tregs) in tumors of treated mice. Ultimately, this results in tumor growth arrest

Our results show that the tumor cell-based vaccine, when used in combination with IL-12 gene therapy, does induce immune response and changes/polarizes the tumor microenvironment from a proangiogenic/immunosuppressive towards an antiangiogenic/immunostimulatory one (Fig. 6).

## Discussion

New therapeutic approaches engage the immunotherapy for cancer treatment (Russo et al. 2014). Tumor immunotherapy aims at restoring the ability to eliminate neoplastic cells through the body’s defense mechanisms (Kozłowska et al. 2013; Mocellin et al. 2004a, b). In clinical studies on advanced melanoma monoclonal antibodies are used. First drug approved by the US Food and Drug Administration was ipilimumab that binds CTLA-4 and blocks the interaction of CTLA-4 with its ligands, CD80 and CD86. Ipilimumab acts via indirect mechanism induced by T lymphocytes in anti-tumor immune response. On the other hand, vemurafenib and dabrafenib inhibit mutations of BRAF V600, which prevents oncogenic activities such as proliferation and evasion of immune response (Olszanski 2014; Russo et al. 2014). A promising alternative is vaccination using whole tumor cells (Chiang et al. 2010). Immunotherapy may be involved in the treatment because tumor cells express tumor-associated antigens, for example, MART1, gp100 and tyrosinase in melanoma (Russo et al. 2014). Vaccines may contain live or dead cancer cells (Kozłowska et al. 2013; Menaria et al. 2013). Whole tumor cells and their lysates contain an entire panel of antigens that can be recognized by DCs, thus markedly increasing the chances of successful therapeutic outcome (Ward et al. 2002). Live cancer cells are weakly immunogenic and release immunosuppressants which block the maturation of DCs [e.g., vascular endothelial growth factor (Peter et al. 2008), transforming growth factor β (Flavell et al. 2010)] or lead to apoptosis of T lymphocytes (Chiang et al. 2010; Ohm et al. 2003). The remnants of dead cancer cells, on the other hand, trigger an immune response. Whole tumor cells as a rich source of antigens expresses the epitopes for CD8^+^ cytotoxic T cells and CD4^+^ T helper (Th) cells. Parallel presentation of antigens both in the context of MHC class I and II molecules contributes to the stronger overall anti-tumor response and long-term immunological memory of CD8^+^ T cells via CD4^+^ Th cells (Chiang et al. 2010). Necrotic cells are phagocytosed by immature DCs, causing the maturation of the latter (Basu et al. 2000; Sauter et al. 2000). This is important insofar as immature DCs stimulate tumor angiogenesis (Ma et al. 2013). Vaccines offer advantages such as low cost, and ease of preparation and storage. They can be administered in a site different from tumor location, so they are useful in treating hard-to-reach malignancies, micrometastases or residual disease. To sum up, tumor cell-based vaccines appear to be a promising tool to induce anti-tumor immune response.

As opposed to the established efficacy of prophylactic vaccines used in treating infectious diseases, the therapeutic effects of anticancer vaccines have remained low in general (Escors 2014). The failure results from the presence of numerous factors supporting tumor growth and escape from immune surveillance (Tabi and Man 2006; Terando et al. 2007). Such factors include uncontrolled proliferation, presence of tumor necrosis and heterogeneous tumor vascular network, release of immunosuppressive cytokines, downregulation of MHC class I molecules, as well as a loss of antigens by cancer cells (Terando et al. 2007). A primary goal of cancer immunotherapy is to elicit CD8^+^ T cells that are able to detect tumor-expressed antigen with high specificity and sensitivity, while limiting damage to normal cells. Moreover, due to Tregs’ potent immunosuppressive function, many emerging strategies aim to augment effector T cell response by the depletion or blockade of Treg lymphocytes in tumors (Savage et al. 2014).

To break immune tolerance, administration of tumor antigens is often combined with the application of immunostimulatory agents (Terando et al. 2007). Enhanced immune response can be obtained using vaccines with various adjuvants. The majority of them are directed at antigen-presenting cells, and they enhance induction of strong cellular immune response by Th1 cells and induction of specific cytotoxic T lymphocytes directed against tumor antigens (Muehlbauer and Schwartzentruber 2003). An example is IL-12, a pleiotropic cytokine inducing a nonspecific (NK, NK-T cells) or specific (CD4^+^ and CD8^+^ T cells) immune responses, as well as showing strong antiangiogenic properties (Del Vecchio et al. 2007; Kilinc et al. 2006; Uemura et al. 2010; Weiss et al. 2007). IL-12 was widely used in preclinical studies, but produced poor outcomes when administered in the form of recombinant protein during clinical studies (Lasek et al. 2014). In our research, we used IL-12 for gene therapy to enhance immune response induced by cell-based vaccine, and analyze the effects of this combination on the polarization of the tumor microenvironment. IL-12 enhances the infiltration of tumor mass by T lymphocytes, macrophages and NK cells (Dickerson et al. 2004). IL-12 also affects the expression of adhesion molecules which take part in directing DCs towards the tumor mass. IL-12 triggers the activation and maturation of DCs (Kim et al. 2006). In addition, IL-12 eliminates regulatory T lymphocytes from the tumor microenvironment, effectively abrogating tumor immunosuppression (Kilinc et al. 2006).

We intended to verify the effectiveness of a tumor cell-based vaccine in combination with IL-12 in inhibiting tumor growth in an experimental murine melanoma model, as well as to study the impact of this combination on the polarization of the tumor microenvironment. There have been conflicting reports concerning the therapeutic benefit of using UV-irradiated cells. Some investigators observed the induction of immunogenic cell death in neoplastic cells (e.g., Obeid et al. 2007), whereas others did not notice such an effect (Fucikova et al. 2011). In our study, we used CAMEL peptide to induce cancer cell death. CAMEL peptide causes mitochondrial swelling and disrupts the mitochondrial membrane, leading to a decrease in intracellular ATP levels, and thus triggering necrotic cell death. Dead tumor cells release alarmins and induce a strong immune response (Chiang et al. 2010). CAMEL-treated tumor cells release factors such as HMGB1 (Smolarczyk et al. 2010). The release of HMGB1 protein causes the inflammation because of the influx of lymphocytes, macrophages, neutrophils and mast cells, activation of defense mechanisms and repair of the affected tissue (Smolarczyk et al. 2010). Furthermore, HMGB1 released during necrotic cell death interacts with Toll-like receptor (TLR)4 on DCs and stimulates the processing and presentation of tumor-derived antigens. TLR4 binds HMGB1 what prevents tumor antigens digestion and facilitates their trafficking to the dedicated antigen-presenting compartment (Chiang et al. 2010). In previous studies, we used CAMEL peptide injected directly into the tumor. This peptide penetrated into cancer cells and caused their necrosis (Smolarczyk et al. 2010). However, cancer cells account only for 30 % of all cells in the tumor (Becker et al. 2013). Therefore, modern therapy has to be targeted at the tumor microenvironment formed by the extracellular matrix, immune cells and tumor blood vessels. In this study, we used CAMEL peptide to design a cell-based vaccine which was then administered to induce an anti-tumor immune response. The purpose of the combination of the cell-based vaccine with IL-12 was to enhance this response and reduce the number of tumor blood vessels necessary for tumor progression.

To this goal, mice were challenged with live B16-F10 melanoma cells and, starting 7 days later, tumor cell-based vaccine was administered contralaterally at weekly intervals (a total of three times). Twenty-four hours after each vaccine administration, pBCMGSNeo/IL-12 plasmid was injected at the same spot. We observed that the vaccine together with IL-12 yielded better tumor inhibitory effects compared to either agent alone. Post-therapeutic analysis showed decreased numbers of tumor blood vessels, considerable necrotic areas, and increased immune cell infiltration in tumor sections from mice treated with the combination regimen. We observed, however, no complete cures following the proposed combination therapy. The decreased effectiveness of our tumor cell-based vaccine may be the result of an existing time span between vaccine effects (treatment initiation), i.e., the appearance of anti-tumor immune effector cells, and the continuing proliferation of cancer cells (Terando et al. 2007). Murine melanoma is a very fast-growing tumor. Immunotherapies require time to maximize their anti-tumor activity, leading to durable response and long disease-stable or disease-free intervals (Olszanski 2014). Additionally, multiple necrotic areas and an abnormal tumor vascular network both limit the contact of tumor-specific cytotoxic T lymphocytes with viable tumor cells (Terando et al. 2007). Also, it ought to be remembered that IL-12-mediated gene therapy was administered (contralaterally) into a non-tumor site. This might weaken the effectiveness of IL-12 treatment (Oshikawa et al. 2001). Intratumoral administration results in increased concentration of IL-12 at the site of endothelial cells’ proliferation, which considerably enhances its antiangiogenic and antineoplastic activity (Dickerson et al. 2004).

Therapy based solely on the use of antiangiogenic factors is not sufficient to inhibit the tumor mass growth. Clinical data show a number of limitations of such therapy: adverse effects, toxicity, acquired drug resistance and aggressive recurrence of tumors after withdrawal of antiangiogenic treatment (Gacche and Meshram 2014). Antiangiogenic therapy is not effective in eliminating the tumor blood vessels arisen in the process of co-option of preexisting normal vessels or vascular mimicry in which neoplastic cells can directly form vessel walls (Moserle et al. 2014). Furthermore, due to antiangiogenic drug-induced therapy, the hypoxia arises. Hypoxia is a major cause of cancer cell invasiveness and metastasis (Azam et al. 2010; Ebos et al. 2009; Keunen et al. 2011; Pàez-Ribes et al. 2009). However, the antiangiogenic therapy has the advantages that may be used in designing combined therapy with cell vaccines. Angiogenic inhibitors have potential anti-tumor activity via re-establishing Treg concentration to a physiological level avoiding autoimmune-mediated side effects, and they do not eliminate activated T cells but inhibit MDSC; they also enhance Th1 response after mitogenic restimulation, and increase the level of tumor-infiltrating T cells. Therefore, antiangiogenic strategies inhibiting tumor-induced immunosuppressive mechanisms create permissive conditions to induce an efficient anti-tumor immune response after vaccination (Terme et al. 2012).

As did Wu et al. (2007), we noted increased levels of T lymphocytes in the cervical lymph nodes of mice treated with the combination therapy, as compared to monotherapies. In this case, we also noted the distinct activation of both nonspecific and specific immune responses. The activation manifests itself as the enhanced infiltration of cells such as CD4^+^ and CD8^+^ T lymphocytes, as well as NK cells, and also as decreased levels of regulatory T lymphocytes in tumors from treated mice. Several studies found a favorable prognostic effect of concurrent infiltration by CD4^+^ and CD8^+^ T cells, as well as NK cell density at the tumor mass (Gutkin and Shurin 2014). NK cells stimulate the maturation of DCs and facilitate adaptive anti-tumor immunity. Indeed, NK cells link innate immunity and adaptive immunity (Zou 2005). NK cells play the key role in the elimination of cancer cells which have lost the ability of MHC expression (Gutkin and Shurin 2014) through the engagement of their activating receptors and the lack of engagement of their inhibitory receptors (Ostrand-Rosenberg 2008). Cytotoxic T lymphocytes (CD8^+^) kill cancer cells after identification of cancer antigens in the context of MHC class I molecules. CD8^+^ T lymphocytes are able to induce the death of cancer cells by cytolytic activity or secretion of effector cytokines such as IFN-γ or TNF-α (Savage et al. 2014). On the other hand, CD4^+^ T lymphocytes form a large fraction of tumor-infiltrating lymphocytes and play an important role in the immune surveillance of the tumor. They are involved in both the induction and effector phase of the anti-tumor immune response (Protti et al. 2014). Two major subpopulations of CD4^+^ T lymphocytes (i.e. Th1 and Th2) are found in tumors. CD4^+^ Th1 lymphocytes elicit anti-tumor activity, both directly by killing cancer cells after identification of cancer antigens in the context of MHC class I molecules and by releasing cytolytic molecules, and indirectly by activating cytokine release from macrophages. CD4^+^ Th2 lymphocytes elicit anti-tumor activity by releasing IL-5 and activation of eosinophils with tumoricidal properties (Protti et al. 2014). The high percentage of Treg cells in various tumors creates the immune suppressive microenvironment that restrains anti-tumor immunity, thus promoting tumor growth (Gutkin and Shurin 2014). Regulatory T cells inhibit the proliferation of CD8^+^ T lymphocytes and the maturation of DCs, and promote tumor angiogenesis (Facciabene et al. 2012; Ostrand-Rosenberg 2008). The accumulation of Treg lymphocytes in tumors is one of the causes of immunosuppressive conditions occurrence (Zou 2005) and the shift in equilibrium between effector and suppressor T cells (Rabinovich et al. 2007; Zou 2005). A decrease in Tregs levels indicates abrogation of the immunosuppressive state in tumors. Reversion (polarization) of the tumor microenvironment by such a drug combination, i.e., stimulation of the immune system to recognize neoplastic cells as foreign, as well as elimination of tumor blood vessels, leads to the arrest of tumor growth (Ciomber et al. 2014; Huang et al. 2012; Jarosz et al. 2013). It seems that the results reported herein implicate such a conversion of tumor milieu.

To summarize, the combination of CAMEL-treated tumor cell-based vaccine and IL-12 does inhibit the growth of B16-F10 murine melanoma experimental tumors. The obtained therapeutic effect is likely caused by tumor microenvironment polarization, which stimulates the immune response and abrogates immunosuppression, and also inhibits the formation of tumor blood vessels. We suppose that combinations of immunomodulation with antiangiogenic agents represent a promising therapeutic approach, useful as a complement to conventional modalities of tumor treatment.
